# An Artificial Intelligence-Driven Multimorbidity Framework Reveals a Shared Metabolic and Immune Core Across Alzheimer’s Disease, Amyotrophic Lateral Sclerosis, and Frontotemporal Dementia

**DOI:** 10.3390/biomedicines14020444

**Published:** 2026-02-16

**Authors:** Meghna R. Iyer, Benjamin Zhao, Xin He, David Camacho, Zihan Wei, Jennifer Deng, Cassie S. Mitchell

**Affiliations:** 1 Laboratory for Pathology Dynamics, Department of Biomedical Engineering, Georgia Institute of Technology & Emory University School of Medicine, Atlanta, GA 30332, USA; 2 Department of Mathematics, Brigham Young University, Provo, UT 84604, USA; 3 Center for Machine Learning at Georgia Tech, Atlanta, GA 30332, USA

**Keywords:** Alzheimer’s disease, amyotrophic lateral sclerosis, frontotemporal dementia, neurodegeneration, machine learning, artificial intelligence, knowledge graph, multimorbidity, neuroinflammation, metabolic dysfunction

## Abstract

**Background/Objectives:** Alzheimer’s disease (AD), amyotrophic lateral sclerosis (ALS), and frontotemporal dementia (FTD) share molecular features yet differ clinically, suggesting underlying systems-level commonalities. We aimed to characterize shared and disease-specific multimorbidity architectures across AD, ALS, and FTD using an artificial intelligence–driven literature-based semantic network. **Methods:** We applied SemNet 2.0, constructed from over 35 million PubMed abstracts, to analyze disease and syndrome (DSYN) and pharmacological substance (PHSU) nodes. Nodes were ranked using HeteSim and mapped to a harmonized 13-category mechanistic ontology. We quantified pairwise disease intersections, ontology-level enrichment, rank similarity, and intersection–disease alignment, and constructed an integrated multimorbidity priority landscape integrating disease-specific and intersection-level hierarchies. **Results:** Across AD, ALS, and FTD, a convergent multimorbidity architecture centered on a shared metabolic and immune core was identified, accompanied by prominent neurobehavioral processes and intermediate systems including gastrointestinal, endocrine, hematological, hepatic, and sensory pathways. Disease-specific signatures shaped distinct vulnerability profiles within this shared structure, including cardiovascular enrichment in AD, neuromuscular and toxin-related pathways in ALS, and coupled neurobehavioral–metabolic features in FTD. PHSU patterns reinforced these findings, with centrally positioned compounds predominantly targeting inflammatory, metabolic, or neuromodulatory processes. **Conclusions:** These findings position AD, ALS, and FTD within a unified, AI-derived multimorbidity framework. This ontology-guided approach provides a computational, hypothesis-generating foundation for multimorbidity-aware biomarker discovery, risk stratification, and cross-disease therapeutic exploration in neurodegenerative disease.

## 1. Introduction

Alzheimer’s disease (AD), amyotrophic lateral sclerosis (ALS), and frontotemporal dementia (FTD) are major neurodegenerative conditions traditionally defined by their clinical and anatomical distinctions. AD is characterized primarily by progressive cognitive impairment, ALS by degeneration of motor neurons and associated respiratory decline, and FTD by alterations in behavior, language, and executive function. Despite these clinical differences, converging evidence from genetics, neuropathology, clinical epidemiology, and molecular biology indicates substantial mechanistic overlap across these disorders [1,2,3]. Shared vulnerability factors include protein aggregation (tau, TDP-43, FUS), mitochondrial dysfunction, oxidative and metabolic stress, synaptic and neurotransmitter dysregulation, and chronic neuroinflammation. Together, these observations suggest that AD, ALS, and FTD may share deeper system-level etiologies that transcend their traditional diagnostic boundaries.

In parallel with these molecular and cellular commonalities, neurodegenerative diseases exhibit complex patterns of comorbidity and antecedent disease. AD is strongly associated with cardiometabolic and vascular conditions such as hypertension, diabetes, obesity, and hypercholesterolemia [4,5]. FTD frequently co-occurs with psychiatric or behavioral syndromes, including depression, anxiety, apathy, or compulsive behavior, reflecting dysfunction in frontal and limbic networks [6,7]. ALS is notable for its unusually low prevalence of many age-related comorbidities, including hypertension, thyroid disease, and hyperlipidemia, raising the possibility of preclinical regulatory differences or compensatory physiological states [8,9]. Moreover, gastrointestinal abnormalities, endocrine disturbances, autoimmune disease, and environmental toxin exposures have been variably implicated across the AD–ALS–FTD spectrum [10,11,12]. Although many comorbidity domains have been independently studied, the field lacks a unified framework that explains how these systems collectively shape shared versus disease-specific vulnerability across neurodegenerative disorders.

Literature-based discovery (LBD) and heterogeneous biomedical knowledge graphs offer a powerful means to address this gap by integrating large-scale, cross-domain biomedical evidence. SemNet 2.0 is an optimized LBD platform that models semantic relatedness between biomedical concepts by mining more than 35 million PubMed abstracts and representing them as a heterogeneous graph of Unified Medical Language System (UMLS) concepts, semantic relationships, and metapaths [13,14]. By using the HeteSim metric to quantify the contextual relatedness between target diseases and source node types, SemNet enables large-scale, literature-driven inference of disease-associated biological processes, comorbidities, and pharmacological agents. Prior SemNet applications have successfully predicted drug repurposing candidates, cross-disease mechanisms, and clinically validated hypotheses [15,16,17].

A previous SemNet analysis by our group demonstrated extensive overlap in amino acid, peptide, and protein (AAPP) molecular signatures across AD, ALS, and FTD [18], motivating a deeper investigation into how systems-level comorbidities and pharmacological associations map onto these molecular relationships. In contrast to the molecular focus of the AAPP study, the present work concentrates on two UMLS source types that directly capture multimorbidity and translational context: diseases and syndromes (DSYN) and pharmacological substances (PHSU). We develop a harmonized 13-category ontology to classify DSYN nodes into mechanistically interpretable systems (e.g., metabolic, immune/inflammatory, cardiovascular, gastrointestinal, endocrine) and use this ontology to quantify shared versus disease-specific multimorbidity across AD, ALS, and FTD. Using DSYN intersections, disease-specific biological hierarchies, rank-based similarity, enrichment patterns, and a unified multimorbidity landscape that juxtaposes disease-specific and intersection-level structures, we map the organization of system-level vulnerability across diseases. PHSU nodes are analyzed as a complementary lens to assess whether pharmacological relevance converges on similar biological systems.

The objective of this study is to delineate the systems-level multimorbidity architecture linking AD, ALS, and FTD; identify the core biological axes that these diseases share; characterize intermediate and disease-specific contributors; and develop a conceptual framework for understanding neurodegeneration through the interaction of metabolic and immune processes with coupled neurobehavioral and environmental factors. This multimorbidity-focused approach offers a generalizable strategy for uncovering cross-diagnostic pathways and for informing future hypothesis generation, risk modeling, and therapeutic exploration across the neurodegenerative disease spectrum.

## 2. Materials and Methods

**Figure 1:**
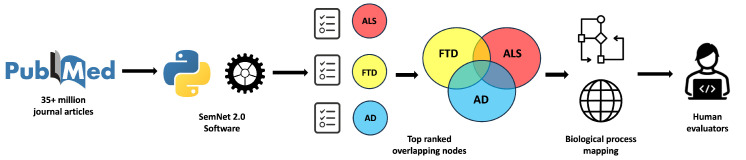
Overall framework for artificial-intelligence-based comparative analysis of multimorbidity across Alzheimer’s disease (AD), amyotrophic lateral sclerosis (ALS), and frontotemporal dementia (FTD). Over 35 million PubMed abstracts were used to construct the heterogeneous biomedical knowledge graph underlying SemNet 2.0. The open-source SemNet 2.0 platform [13], together with its post-visualization tool CompositeView [19], was used to identify and rank Unified Medical Language System (UMLS) disease and syndrome (DSYN) and pharmacological substance (PHSU) nodes associated with AD, ALS, FTD, and their pairwise intersections using HeteSim-based semantic relatedness; pairwise disease intersections were defined by shared DSYN nodes exceeding a harmonic mean threshold (HM ≥ 0.5) across disease-specific normalized HeteSim scores, emphasizing balanced cross-disease relevance. DSYN nodes were mapped to 13 mechanistically interpretable biological process categories using a combination of cross-domain text mining, natural language processing, and a large language model. To validate and refine these mappings, three human evaluators independently reviewed category assignments for high-importance nodes via full-text article inspection, with high inter-rater reliability (Cohen’s κ=0.88). This human validation step was implemented to minimize the inadvertent inclusion of disease-defining or symptom-level features and to ensure that node labels reflected system-level multimorbidity rather than disease-specific manifestations.

To systematically characterize multimorbidity structure across Alzheimer’s disease (AD), amyotrophic lateral sclerosis (ALS), and frontotemporal dementia (FTD), we employed a multi-stage analytical pipeline integrating semantic network inference, ontology mapping, and cross-disease comparative analyses as shown in Figure 1. First, disease-associated DSYN and pharmacological substance (PHSU) nodes were identified and ranked using the SemNet 2.0 framework. These nodes were then mapped to a harmonized mechanistic ontology to enable system-level interpretation. Subsequent analyses quantified pairwise disease intersections, disease-specific hierarchies, and cross-disease rank similarity, culminating in an integrated priority landscape that captures shared, intermediate, and disease-specific biological vulnerabilities.

### 2.1. SemNet 2.0 Knowledge Graph and HeteSim Ranking

SemNet 2.0 constructs a heterogeneous biomedical knowledge graph from semantic triples mined from PubMed, where nodes correspond to UMLS concepts and edges represent semantically typed relationships such as “treats,” “affects,” or “is associated with” [13,14]. For this study, the target nodes were AD, ALS, and FTD. For each target disease, we performed SemNet simulations with DSYN and PHSU as source node types. Search depth was set to 2 and metapath length to 3, parameters previously shown to balance recall and interpretability [13]. HeteSim scores were computed using deterministic mean aggregation across all metapaths linking each source node to the target disease. Scores were normalized by subtracting the mean, scaling to unit variance, and converting to percentile ranks, facilitating cross-simulation comparisons. CompositeView, a visualization and score aggregation tool, was used to generate two-dimensional semantic neighborhood maps for high-importance DSYN and PHSU nodes. This projection places semantically related concepts in close proximity, allowing qualitative and aggregated quantitative assessment of shared versus disease-specific node clustering across AD, ALS, and FTD. For more details on the SemNet 2.0 [13] and CompositeView [19] pipeline, please see our prior work [18].

### 2.2. Mapping DSYN Nodes to a 13-Category Mechanistic Ontology

DSYN nodes returned by SemNet include diseases, syndromes, and clinical conditions that may represent comorbidities, antecedent disease, risk factors, complications, or disease-related manifestations. To make these results mechanistically interpretable, we mapped all DSYN nodes to a harmonized 13-category ontology. DSYN nodes were mapped to 13 mechanistically interpretable biological process categories using a combination of cross-domain text mining, natural language processing, and a large language model to generate initial category assignments. To validate and refine these mappings, three human evaluators independently reviewed the biological category assignments for high-importance nodes using full-text article inspection. Inter-rater reliability across evaluators was high (Cohen’s κ=0.88), indicating strong agreement prior to consensus adjudication. This additional human evaluation step was implemented to minimize the inadvertent injection of disease-defining or symptom-level features into the multimorbidity framework and to ensure that category assignments reflected system-level biological processes rather than disease-specific manifestations.

- **Metabolic**: Disorders involving energy metabolism, mitochondrial function, glucose and lipid regulation, insulin signaling, or systemic metabolic homeostasis (e.g., diabetes, metabolic syndrome, dyslipidemia).
- **Immune/Inflammatory/Infectious**: Conditions reflecting innate or adaptive immune activation, autoimmune disease, chronic inflammation, infection-associated inflammatory sequelae, or systemic immune dysregulation.
- **Cardiovascular**: Diseases affecting cardiac function, vascular tone, blood pressure regulation, atherosclerosis, coronary artery disease, or cerebrovascular circulation.
- **Other/Neuro-Psych**: Cognitive, behavioral, mood, and neuropsychiatric syndromes, including dementia, cognitive impairment, personality or behavioral changes, mood disorders, and neurotransmitter-related disturbances. Other/Neuro-Psych may reflect disease-adjacent cognitive and behavioral phenomena rather than an independent comorbid disease burden.
- **Sensory (eyes, ears, throat; EET)**: Disorders involving sensory organs or cranial structures, including visual, auditory, vestibular, olfactory, and upper-airway systems.
- **Gastrointestinal (GI)**: Conditions affecting digestive tract function, gut inflammation, microbiome-related disturbances, malabsorption, or gastrointestinal motility.
- **Endocrine**: Disorders of hormonal signaling and endocrine glands (e.g., thyroid, adrenal, pituitary, pancreatic endocrine axis), including hormone imbalance and endocrine-driven metabolic disturbance.
- **Hematological**: Diseases of blood cells, coagulation, bone marrow function, anemia, or clotting disorders.
- **Dermatological**: Skin and integumentary conditions reflecting inflammatory, autoimmune, infectious, or degenerative processes.
- **Liver**: Hepatic disorders related to metabolism, detoxification, inflammation, bile processing, or drug-induced liver injury.
- **Kidney**: Renal diseases related to filtration, electrolyte balance, renal inflammation, or chronic kidney dysfunction.
- **Musculoskeletal/Orthopedic**: Disorders of muscle, bone, joints, or connective tissue, including neuromuscular involvement that may intersect with motor-system vulnerability.
- **Environmental Toxins**: Conditions linked to exposure to toxicants such as heavy metals, pesticides, solvents, or other environmental or occupational hazards.

### 2.3. Merging Neurological and Psychiatric DSYN Categories into Other

DSYN nodes were provisionally separated into “neurological antecedent” and “psychiatric or mental health” categories. However, inspection showed these sets were heavily enriched in disease-defining features of AD and FTD (e.g., dementia, cognitive impairment, aphasia, personality changes, mood disorders) rather than an independent comorbidities. Moreover, the SemNet 2.0 knowledge nodes do not enable specific splitting of these neurological nuances. To avoid duplicating the disease phenotype as a comorbidity, they were merged into a single Other/Neuro-Psych category representing all explicit neurological, cognitive, behavioral, mood, and neuropsychiatric processes, including both antecedent and disease-adjacent phenomena.

### 2.4. Defining DSYN Intersections via Harmonic-Mean Thresholds

Pairwise DSYN intersections were calculated for each disease pair (AD–ALS, AD–FTD, ALS–FTD). For each DSYN node that appeared in both disease-specific DSYN outputs, we computed the harmonic mean (HM) of its normalized HeteSim scores:

$$\mathrm{HM}=\frac{2}{\frac{1}{H_{d1}}+\frac{1}{H_{d2}}}$$

The harmonic mean was selected to emphasize balanced relevance across diseases, as it penalizes nodes that are highly associated with one disease but weakly associated with the other. In contrast to arithmetic averaging, the HM ensures that intersection nodes reflect shared semantic importance rather than dominance by a single disease.

A threshold of HM≥0.5 was used to define jointly important DSYN nodes. Because HeteSim scores were normalized to the unit interval, this criterion retains nodes that exhibit at least moderate semantic relatedness to *both* diseases, corresponding to the upper half of the balanced relevance range. This threshold therefore provides an interpretable and conservative operational definition of cross-disease multimorbidity while maintaining sufficient node counts for stable ontology-level analyses.

Each intersection DSYN node was assigned to a single ontology category. For each intersection, we computed category-level node counts, within-intersection percentages (normalized to 100% per intersection), and Z-score enrichment relative to the global DSYN category distribution across all three diseases.

### 2.5. Disease-Specific DSYN Hierarchies and Cross-Disease Similarity

For each disease, we aggregated DSYN normalized HeteSim scores by ontology category and ranked categories from 1 (highest importance) to 13 (lowest). These disease-specific hierarchies capture how each disease preferentially engages different biological systems. Cross-disease similarity was quantified using Spearman rank correlation and Kendall Tau for each pair of diseases (AD vs. ALS, AD vs. FTD, ALS vs. FTD).

### 2.6. Intersection–Disease Alignment and Rank Differences

To evaluate how intersection biology related to disease-specific hierarchies, we:

1. Computed Spearman correlations between each intersection’s category rank vector and its parent diseases’ rank vectors.
2. Calculated disease–intersection rank differences (disease rank minus intersection rank) per category. Negative values indicate categories prioritized more within the disease than within the intersection, whereas positive values indicate categories that are up-weighted in intersection space relative to disease-specific hierarchies.

### 2.7. Integrative Ranking Across Diseases

To summarize system-level importance across Alzheimer’s disease (AD), amyotrophic lateral sclerosis (ALS), and frontotemporal dementia (FTD), we employed two complementary, non-aggregative strategies. First, disease-specific and intersection-level category ranks were jointly organized into an integrated DSYN priority landscape by converting ordinal ranks to rank-based priority scores (e.g., 15 minus rank) for visual comparability. This approach preserves the ordinal nature of rank data and enables side-by-side comparison of system-level prominence across diseases and intersections without averaging ranks across diseases.

Second, for descriptive cross-disease summarization only, we computed an integrative category ranking using a Borda aggregation across disease-specific category ranks. This ordinal aggregation preserves rank information and avoids mathematically inappropriate averaging of ordinal values. The resulting integrative rankings were used solely as a numerical summary of cross-disease prioritization and were not used to generate figures or downstream statistical analyses.

### 2.8. PHSU Analysis

PHSU nodes represent pharmacological substances, including therapeutic agents, nutraceuticals, natural products, and toxicants. High-importance PHSU nodes for each disease were analyzed by grouping substances into broad mechanistic classes (e.g., anti-inflammatory, antioxidant, neuromodulatory, cardiometabolic, antimicrobial). We qualitatively examined proximity patterns across diseases (e.g., compounds closer to ALS versus FTD or AD) and interpreted these patterns alongside the DSYN-based multimorbidity architecture.

## 3. Results

This study used a semantic-network-based approach to characterize multimorbidity patterns across Alzheimer’s disease (AD), amyotrophic lateral sclerosis (ALS), and frontotemporal dementia (FTD). High-importance disease and syndrome (DSYN) nodes and pharmacological substance (PHSU) nodes were extracted from SemNet 2.0 simulations and mapped to a harmonized 13-category mechanistic ontology. We first provide a visual overview of the semantic neighborhoods that define DSYN and PHSU relationships across the three diseases, establishing a conceptual foundation for subsequent quantitative analyses. We then examine pairwise DSYN intersections, disease-specific biological hierarchies, rank-based similarity between diseases, and intersection–disease alignment. Finally, we present an integrated DSYN priority landscape that jointly visualizes system-level contributions across diseases and intersections, followed by analyses of detailed comorbidity clusters and pharmacological patterns to interpret how the ontology-derived multimorbidity architecture reflects known epidemiologic, clinical, and mechanistic features of AD, ALS, and FTD.

### 3.1. Overview of Semantic Neighborhood Structure Revealed by CompositeView

Before evaluating multimorbidity patterns through ontology mapping, intersections, and rank-based analyses, it is important to visualize the overall semantic structure of how diseases, comorbidities, and pharmacological agents relate to one another in the literature-derived biomedical knowledge graph. To provide this high-level orientation, we used SemNet 2.0’s CompositeView, a two-dimensional projection that places semantically related nodes close together while maintaining disease specificity for those concepts uniquely associated with AD, ALS, or FTD.

**Figure 2:**
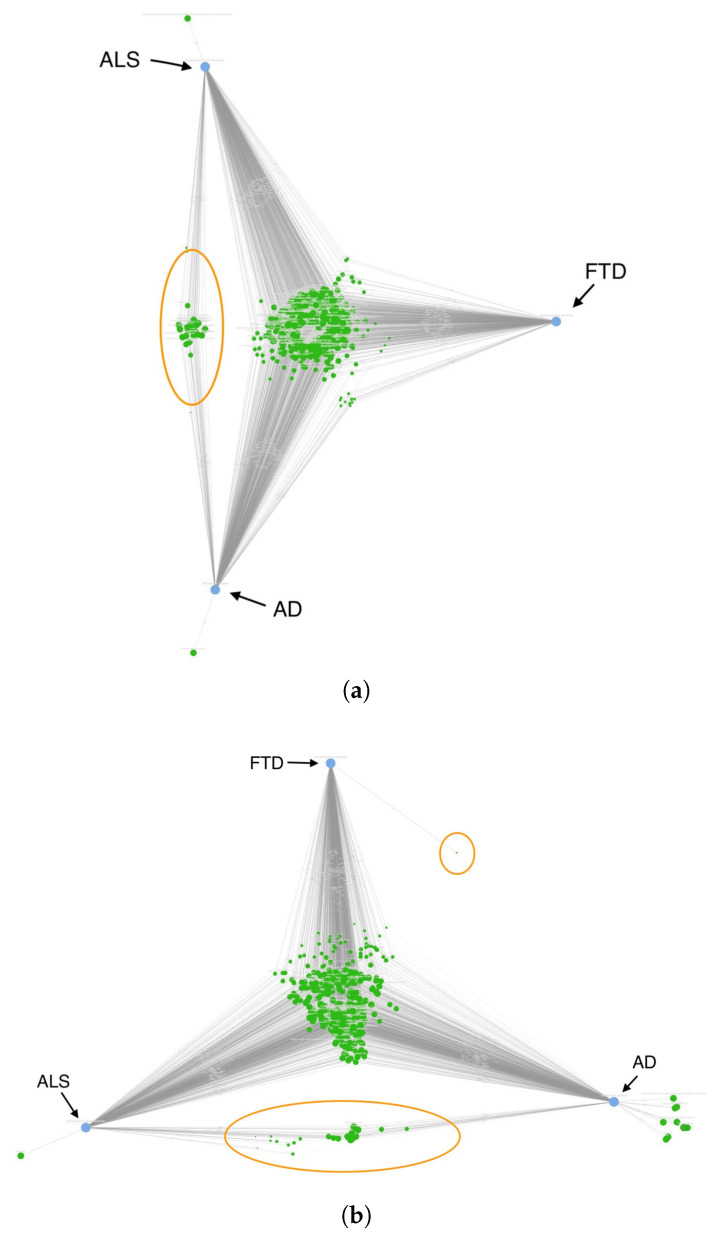
SemNet 2.0 was used to examine intersections among AD, FTD, and ALS using the Unified Medical Language System (UMLS) disease and syndrome (DSYN) and pharmacological substances (PHARM) node types. CompositeView was used to examine the shared node spaces. (**a**) DSYN node type space for AD, FTD, and ALS. The circle illustrates conglomerates of nodes shared by AD and ALS but not FTD. (**b**) PHARM node type space for AD, FTD, and ALS. The circles illustrate conglomerates of nodes shared by FTD and AD (upper right) or ALS and AD (bottom).

Figure 2a shows the CompositeView distribution of high-importance DSYN (disease and syndrome) nodes for AD, ALS, and FTD. The dense central cluster reflects a large set of DSYN nodes that are jointly relevant to all three diseases, demonstrating that AD, ALS, and FTD inhabit a shared conceptual and clinical space with extensive overlap in comorbidities, antecedent conditions, and disease-adjacent syndromes. Smaller peripheral clusters correspond to DSYN nodes that preferentially align with a single disease, such as toxin-associated or neuromuscular conditions in ALS or cardiovascular and dementia-related descriptors in AD. This structural map illustrates why ontology-based categorization and pairwise intersection analyses are essential: although the diseases share many semantic neighbors, the nature and composition of these neighbors differ, and quantitative analyses are required to disentangle shared from disease-specific contributors.

Figure 2b shows the corresponding CompositeView distribution for high-importance PHSU (pharmacological substance) nodes. Here too, a substantial overlap is observed, with many compounds positioned in shared regions between diseases. These typically include anti-inflammatory, antioxidant, neuromodulatory, antimicrobial, or metabolic-modulating substances that have been studied across multiple neurodegenerative contexts. Compounds located nearer to a specific disease—such as cardioactive agents closer to AD or toxin-related substances closer to ALS—suggest pharmacological relevance that mirrors DSYN-based multimorbidity patterns.

**Figure A1:**
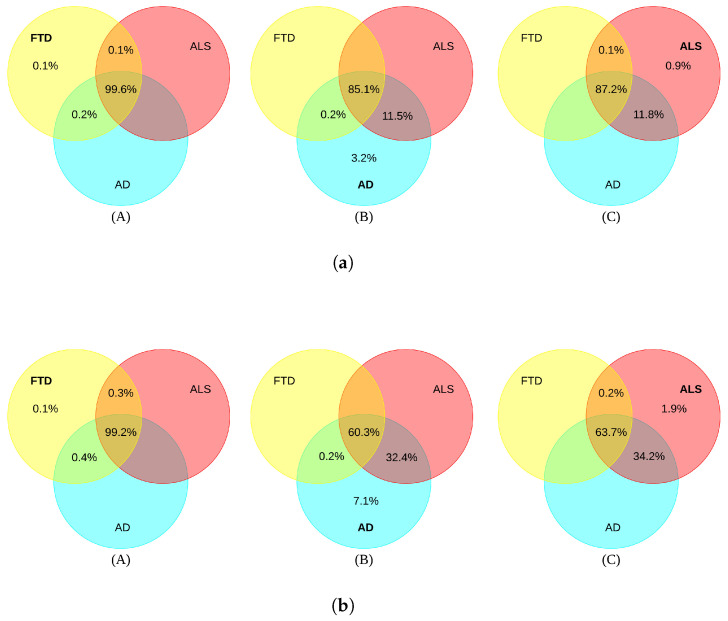
Venn diagrams illustrating the overlap of high-importance nodes among AD, ALS, and FTD. The Venn diagram of three diseases (AD, ALS and FTD) illustrating intersections and unions for PHSU (Pharmacological Substance) node type. FTD represents the light yellow circles; AD represents the aqua blue circles; ALS represents the light red circles. Intersections are shown in percentages for each disease. (**a**) DSYN (Diseases and Syndromes) node overlap demonstrates that a substantial fraction of high-importance DSYN concepts are shared among all three diseases, with smaller disease-specific subsets. (A) FTD; (B) AD; (C) ALS. (**b**) PHSU (pharmacological substance) node overlap similarly shows extensive shared pharmacological neighborhoods, consistent with the CompositeView projections in the main text. (A) FTD; (B) AD; (C) ALS.

As complementary quantitative summaries, Figure A1 includes DSYN and PHSU Venn diagrams that numerically illustrate the degree of node overlap among AD, ALS, and FTD. These diagrams corroborate the shared-space patterns observed in the CompositeView projections, further supporting the presence of extensive common semantic neighborhoods with smaller disease-specific subsets.

Together, the DSYN and PHSU CompositeViews provide a visual foundation for the analyses that follow. They demonstrate: (i) that a substantial portion of the SemNet-derived disease- and drug-related conceptual space is shared among AD, ALS, and FTD; and (ii) that meaningful disease-specific clusters also exist. This motivates a systematic, ontology-guided approach to quantify shared versus disease-specific mechanisms and to map multimorbidity architecture across these neurodegenerative diseases. The next subsections therefore examine DSYN intersections, category-level patterns, rank correlations, integrated priority landscapes, and detailed comorbidity themes using this conceptual foundation as context.

### 3.2. DSYN Multimorbidity Intersections Highlight a Shared Metabolic–Immune Axis

**Figure 3:**
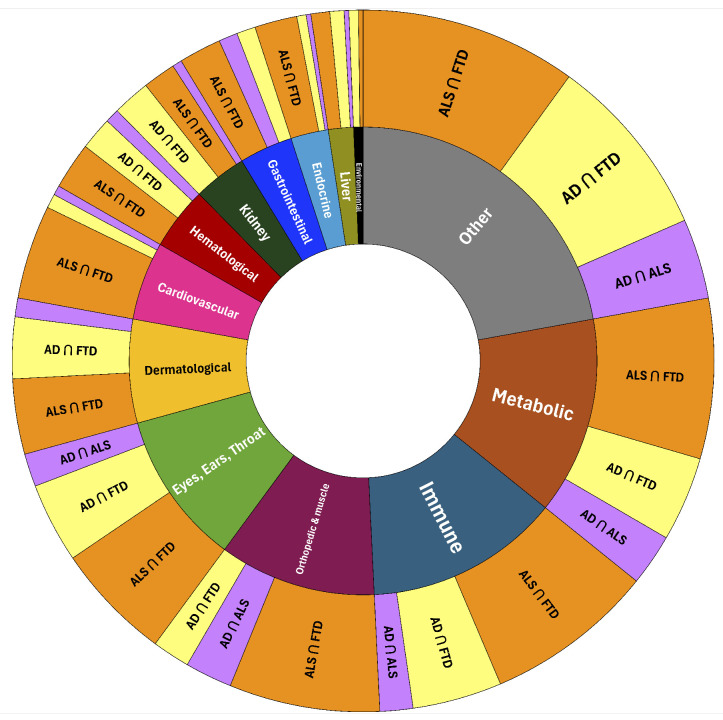
Sunburst representation of DSYN-based multimorbidity intersections among AD, ALS, and FTD. The figure shows the distribution of high-importance DSYN nodes across the 13-category ontology for each pairwise intersection (AD∩ALS, AD∩FTD, ALS∩FTD), highlighting a dominant contribution of Metabolic and Immune/Inflammatory systems across all intersections, with additional representation from the Other/Neuro-Psych category and intermediate system-level domains.

**Figure 4:**
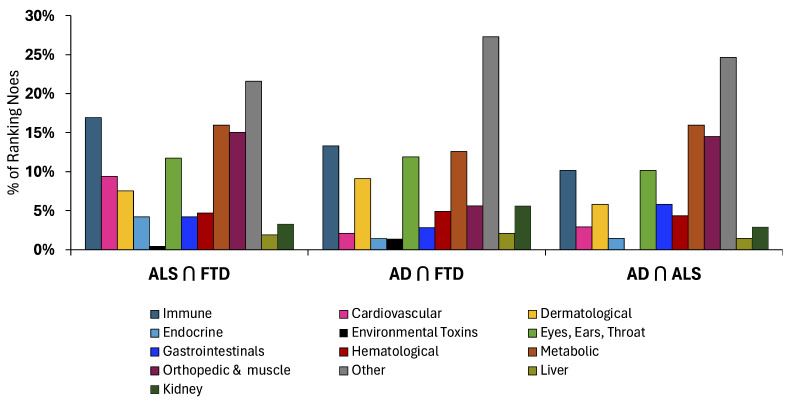
Category-wise percentage of high-importance DSYN nodes within each pairwise intersection of AD, ALS, and FTD. Each bar represents the fraction of intersection nodes assigned to a given ontology category, normalized to 100% per intersection. Metabolic, Immune/Inflammatory, and Other/Neuro-Psych categories consistently account for the largest fractions across intersections, with additional contributions from Musculoskeletal/Orthopedic and Sensory (EET) systems.

**Figure 5:**
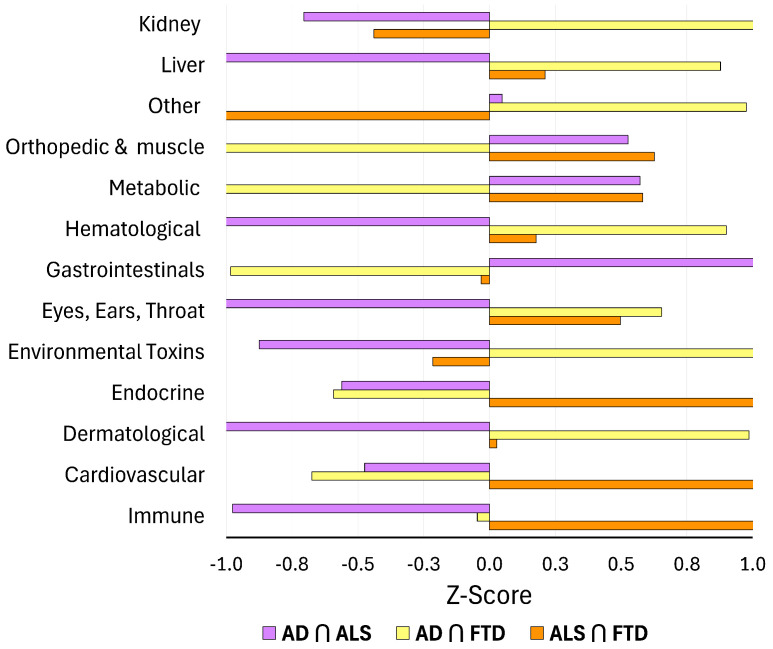
Z-score enrichment of DSYN ontology categories within intersections relative to the global DSYN category distribution across all three diseases. Positive Z-scores indicate categories that are over-represented in a given intersection, whereas negative values indicate under-representation. Metabolic and Immune/Inflammatory categories show strong positive enrichment across intersections, while Other/Neuro-Psych categories are consistently elevated, reflecting a mixture of neuropsychiatric comorbidities and disease-adjacent phenomena. Environmental Toxins and Dermatological categories are generally under-represented.

Figure 3 provides a high-level visualization of the biological systems represented within each DSYN intersection. The sunburst diagram highlights the overall structure and relative balance of ontology categories contributing to multimorbidity, illustrating that all three disease pairs share a broadly similar systems-level profile dominated by Metabolic and Immune/Inflammatory contributions, with elevated Other/Neuro-Psych categories reflecting disease-adjacent cognitive, behavioral, and neuropsychiatric phenomena rather than an independent comorbid disease burden. Qualitative differences are still evident across intersections: the AD∩FTD intersection shows the most pronounced representation, ALS∩FTD displays comparatively stronger contributions from Musculoskeletal/Orthopedic and Sensory (EET) systems, and AD∩ALS shows a more diffuse pattern consistent with weaker overall similarity between the two diseases. Categories such as Liver, Kidney, Hematological, and Dermatological processes remain minimally represented across all intersections. This qualitative overview motivates the more detailed quantitative comparisons provided in the subsequent percentage-based (Figure 4) and enrichment-based analyses (Figure 5).

To quantify these qualitative patterns, Figure 4 presents the percentage of high-importance DSYN nodes assigned to each ontology category within the AD∩ALS, AD∩FTD, and ALS∩FTD intersections. Across all three pairs, Metabolic, Immune/Inflammatory, and Other/Neuro-Psych categories consistently account for the largest proportions of intersection nodes, indicating that disturbances in energy metabolism, chronic inflammatory signaling, and cognitive–behavioral regulation constitute the major shared multimorbidity axes linking these neurodegenerative diseases. Musculoskeletal/Orthopedic and Sensory (EET) pathways contribute secondary, but nontrivial, fractions across intersections, suggesting supporting roles in multimorbidity structure. In contrast, categories such as Liver, Kidney, Hematological, and Dermatological systems exhibit only minor representation, implying more limited cross-disease relevance. Although the overall composition patterns are broadly similar across all intersections, subtle differences in category proportions highlight that each disease pair has a distinct multimorbidity profile. These differences are further clarified by the Z-score enrichment analysis shown in Figure 5, which identifies categories that are disproportionately represented relative to their global baseline prevalence.

Z-score enrichment analyses, computed relative to the global DSYN category distribution across all three diseases, further clarified which biological systems are disproportionately represented within multimorbidity space (Figure 5). As anticipated based on ontology construction, Other/Neuro-Psych categories exhibited elevated enrichment due in part to the inclusion of disease-adjacent cognitive, behavioral, and neuropsychiatric phenomena (see Methods). In contrast, Metabolic and Immune/Inflammatory categories demonstrated the strongest and most consistent positive enrichment across all intersections, reinforcing their roles as dominant, cross-cutting mechanisms linking AD, ALS, and FTD.

Neuropsychiatric and behavioral processes nevertheless remain prominently represented within shared-node space, indicating that these features co-occur with systemic multimorbidity patterns rather than functioning solely as disease-specific manifestations. In contrast, the Environmental Toxins and Dermatological categories showed pronounced negative enrichment, suggesting that although these systems may be prominent within individual diseases—such as toxin-related DSYN nodes in ALS—they do not constitute core components of shared multimorbidity architecture. Intermediate categories, including Gastrointestinal, Endocrine, Hematological, and Sensory (EET) systems, exhibited modest and intersection-dependent enrichment patterns, consistent with secondary but biologically meaningful contributions observed in earlier analyses. Collectively, the Z-score results highlight that the most strongly enriched systems across intersections mirror the biological axes identified in both the sunburst visualizations and percentage distributions, providing quantitative support for a shared metabolic–immune multimorbidity core.

Together, these findings identify metabolic dysfunction, immune/inflammatory dysregulation, and neurobehavioral systems as a shared multimorbidity core across AD, ALS, and FTD, with musculoskeletal and sensory systems acting as frequent adjuncts.

### 3.3. Disease-Specific DSYN Hierarchies Show Asymmetric Similarity Across Diseases

Disease-specific DSYN hierarchies revealed that AD and FTD were most similar in how they prioritized ontology categories, followed by AD and ALS, with ALS and FTD being the least similar. AD and FTD both assigned high priority to Metabolic and Immune categories. ALS also prioritized these systems but uniquely emphasized the Environmental Toxins and Musculoskeletal/Neuromuscular categories, distinguishing its DSYN profile from AD and FTD [2,3,12].

Spearman and Kendall Tau correlations reflected these patterns quantitatively, with the highest correlations observed for AD–FTD, moderate for AD–ALS, and the lowest for ALS–FTD. These relationships align with known molecular and clinical overlaps: AD and FTD share cortical degenerative patterns and, in some cases, overlapping genetic and cognitive phenotypes, while ALS is more distinct, except where it overlaps with FTD in ALS–FTD continuum syndromes [20,21,22,23].

### 3.4. FTD Exerts Disproportionate Influence on DSYN Intersection Hierarchies

**Figure 6:**
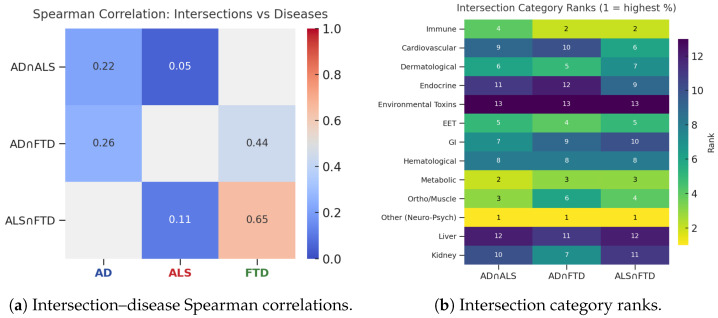
Alignment between DSYN intersection hierarchies and disease-specific DSYN hierarchies. (**a**) Spearman correlation coefficients between each intersection (AD∩ALS, AD∩FTD, ALS∩FTD) and the corresponding disease-specific category rankings. (**b**) Heatmap of category ranks within each intersection (1 = highest percentage).

The extent to which each DSYN intersection recapitulates the biological prioritization of its constituent diseases is shown in Figure 6. These patterns demonstrate asymmetric alignment, with the AD∩FTD and ALS∩FTD intersections more closely tracking the FTD hierarchy, whereas the AD∩ALS intersection exhibits comparatively diffuse similarity.

Quantitative intersection–disease comparisons confirmed this asymmetry: for both AD–FTD and ALS–FTD intersections, category rankings aligned more strongly with the FTD DSYN hierarchy than with the corresponding AD or ALS hierarchies. By contrast, the AD–ALS intersection showed weaker alignment with either disease, indicating less coherent shared prioritization in the absence of FTD.

Together, these results position FTD as a “bridge” disease in multimorbidity space. FTD’s strong emphasis on metabolic and neurobehavioral categories appears to propagate into shared-node DSYN intersections, consistent with its established role along the ALS–FTD spectrum and with mixed AD–FTD phenotypes observed in clinical practice [23,24,25]. This bridging role suggests that FTD occupies an intermediate systems-level position linking cortical neurodegeneration more typical of AD with motor-system vulnerability characteristic of ALS.

### 3.5. Disease–Intersection Rank Differences Distinguish Shared Versus Disease-Specific Mechanisms

**Figure 7:**
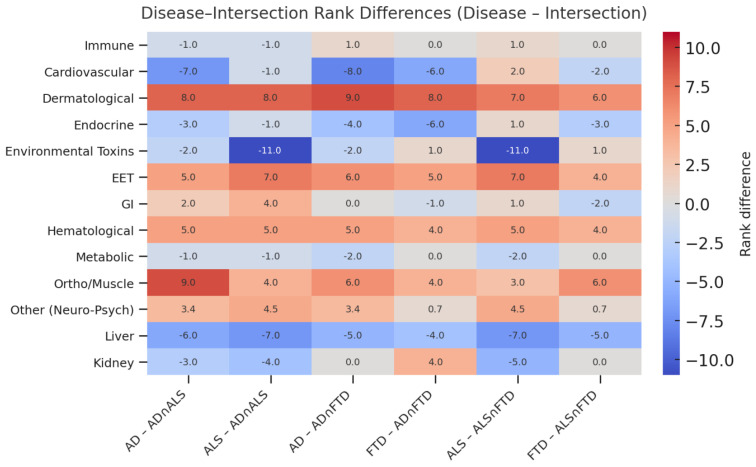
Disease–intersection rank differences across DSYN categories. Values represent disease rank minus intersection rank. Negative values indicate categories prioritized more highly within the disease than in the corresponding intersection, whereas positive values indicate categories disproportionately up-weighted in shared-node space.

Disease–intersection rank differences provided insight into which biological systems are enriched within diseases versus intersections. Disease–intersection rank differences across DSYN categories are shown in Figure 7, which illustrates how each disease contributes uniquely to its intersection profile. Cardiovascular pathways were relatively up-weighted in AD; toxin- and neuromuscular-related pathways were more prominent in ALS; and Other/Neuro-Psych and Musculoskeletal categories tended to be elevated within intersection space compared to disease-specific hierarchies.

Cardiovascular categories tended to be ranked higher within AD than in intersections, indicating that cardiovascular DSYN nodes are more central to AD-specific vulnerability than to cross-disease multimorbidity [4,26,27]. Environmental Toxin categories, including exposure to solvents, heavy metals, and toxin-associated neuropathies, were more strongly prioritized within ALS than in intersections, suggesting that environmental exposures are comparatively disease-specific and do not constitute a primary shared multimorbidity axis [8,12,28].

By contrast, Other/Neuro-Psych and Musculoskeletal categories were frequently more prominent in intersections than in disease-specific hierarchies, indicating that they play a particularly important role in shared multimorbidity space. Gastrointestinal, Endocrine, Hepatic, Hematological, and Sensory/EET categories showed more mixed or intermediate patterns, suggesting context-dependent contributions.

### 3.6. Integrated DSYN Priority Landscape and Intermediate Systems

**Figure 8:**
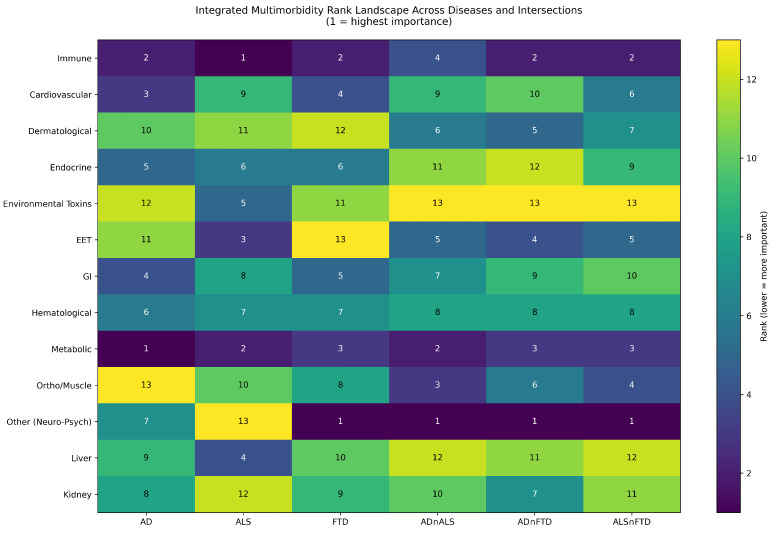
Integrated DSYN-based multimorbidity landscape across AD, ALS, FTD, and their pairwise intersections. Each cell reports the ordinal rank of a biological ontology category within a disease or intersection (1 = highest importance). Disease-specific ranks are derived from category-level DSYN hierarchies, and intersection ranks are derived from category-level intersection hierarchies. Color intensity reflects relative rank-based priority within each entity. This integrated visualization enables direct comparison of shared and disease-specific system-level prioritization without aggregating or averaging ordinal ranks across diseases.

To synthesize disease-specific and intersection-level findings, we constructed an integrated DSYN priority landscape by jointly visualizing disease-specific and intersection-level category ranks, converted to rank-based priority scores (e.g., 15 minus rank) for comparability. Figure 8 presents a unified DSYN-based multimorbidity landscape that juxtaposes disease-specific and intersection hierarchies, revealing the overarching architecture of shared and divergent vulnerabilities. This consolidated view highlights a shared metabolic and immune core across AD, ALS, and FTD, while also delineating intermediate systems and disease-specific peaks that shape distinct multimorbidity signatures. Cardiovascular pathways are more strongly prioritized in AD, and toxin-related and neuromuscular pathways are more strongly prioritized in ALS, while Musculoskeletal categories tend to be up-weighted within intersection space relative to disease-specific hierarchies. This unified heatmap enables direct comparison of system-level prominence across all six entities without aggregating ordinal ranks across diseases.

To provide a numerical complement to this visual comparison, we examined disease-specific category rankings alongside a descriptive cross-disease summary of category importance. Table 1 reports disease-specific rankings and a Borda-based ordinal summary across AD, ALS, and FTD, offering a compact numerical overview of system-level prominence without driving downstream statistical analyses.

Across the landscape, Metabolic and Immune/Inflammatory categories consistently occupy the highest priority positions, forming a shared multimorbidity core across all three diseases and their intersections. Other/Neuro-Psych categories are also prominently represented across disease-specific and intersection contexts, reflecting the close coupling of neurobehavioral processes with systemic multimorbidity rather than an independent core driver.

A second group of categories exhibits intermediate and context-dependent priority, including Gastrointestinal (GI), Endocrine, Hematological, Liver, and Sensory (ears, eyes, throat; EET) systems. These categories rarely dominate disease-specific DSYN hierarchies individually, yet they frequently demonstrate moderate elevation in intersection contexts, indicating that they may function as modulators rather than primary drivers of multimorbidity. Mechanistically, these systems interface with metabolic and immune regulation—for example, through microbiome-mediated immune activation, hormonal–metabolic interactions, or systemic inflammatory load—thereby shaping disease expression without defining it.

In contrast, certain categories show disease-specific prominence with limited contribution to shared multimorbidity. Cardiovascular DSYN nodes are strongly prioritized in AD but not within DSYN intersections, consistent with the established role of vascular risk factors as AD-specific determinants rather than cross-disease mechanisms. Environmental Toxins and Musculoskeletal/Orthopedic categories are distinctly elevated in ALS-specific hierarchies but contribute minimally in intersection profiles, reflecting ALS’s particular susceptibility to exposure-related and neuromuscular factors. Dermatological categories remain consistently low priority across all diseases and intersections, suggesting limited relevance to multimorbidity in this neurodegenerative context.

Overall, the integrated DSYN priority landscape reinforces the emerging theme that multimorbidity in AD, ALS, and FTD is structured around a shared metabolic and immune axis, supplemented by intermediate systems and modulated by disease-specific vulnerabilities. This landscape provides a coherent framework for interpreting the detailed DSYN comorbidity clusters and pharmacological patterns presented in subsequent sections.

### 3.7. Grounding the Multimorbidity Architecture to Common Antecedent or Comorbid Risk Factors

The ontology-based analyses and integrated multimorbidity landscape identified a shared set of biological systems contributing to Alzheimer’s disease (AD), amyotrophic lateral sclerosis (ALS), and frontotemporal dementia (FTD). To provide mechanistic context for these system-level patterns, we examined representative subsets of disease-associated DSYN nodes derived from SemNet. These node subsets, summarized in the Appendix A, capture commonly cited comorbid conditions and risk factors and offer mechanistic detail that complements the higher-level ontology rankings. Together, they reinforce the ontology-driven results while illustrating how specific disease- and system-level processes map onto shared and disease-specific multimorbidity architecture.

#### 3.7.1. Diabetes

Multiple DSYN nodes across all diseases relate to diabetes and metabolic dysregulation, consistent with the prominent role of Metabolic ontology categories in both disease-specific and intersection analyses. Epidemiologic data demonstrate strong links between type 2 diabetes, insulin resistance, and increased risk of AD and FTD, whereas the association with ALS is more complex: early-life diabetes may increase ALS susceptibility, while later-life diabetes may confer a modest protective effect [29,30,31]. Mechanistic pathways implicated in these associations include impaired glucose metabolism, mitochondrial stress, oxidative phosphorylation deficits, vascular compromise, and chronic inflammation [32,33]. These processes align closely with the metabolic–immune axis highlighted in the DSYN intersection, enrichment, and priority landscape analyses. Similarly, Table A1 provides representative DSYN nodes and HeteSim scores for diabetes-related conditions, illustrating how specific metabolic disease subtypes map onto the broader multimorbidity architecture described in this section.

#### 3.7.2. Common Cardiovascular Risk Factors

Cardiovascular disease and cardiovascular-associated DSYN nodes—including hypertension, hypercholesterolemia, obesity, and coronary disease—were particularly prominent for AD and variably present for ALS and FTD, reflecting their placement within the Cardiovascular ontology category. Hypertension and hyperlipidemia are well-established risk factors for cognitive decline and AD progression [34,35], while obesity contributes to chronic systemic inflammation via cytokine activation [26,27]. In ALS, antecedent cardiovascular conditions show mixed effects: several studies indicate lower overall cardiovascular comorbidity prevalence among ALS patients, yet specific risk factors such as hypertension or hyperlipidemia may delay disease onset or, in some subgroups, shorten survival [8,9,36]. These distinctions are reflected in the DSYN landscape, where cardiovascular contributions are comparatively AD-specific and show weaker prominence within multimorbidity intersections. To complement these thematic summaries, Table A2 provides representative DSYN nodes and HeteSim scores illustrating how individual cardiovascular-risk-related conditions map onto the multimorbidity architecture described in this subsection.

#### 3.7.3. Gastrointestinal and Gut–Brain Axis Involvement

DSYN nodes linked to gastrointestinal inflammation, infection, dysbiosis, and functional GI disorders map to the Gastrointestinal ontology category and mirror growing evidence for a microbiome–gut–brain axis in neurodegeneration [10,11,37]. GI disturbances can alter microbial composition, immune signaling, and the production of neuroactive compounds such as serotonin, GABA, and short-chain fatty acids. Aberrant GI function has been observed in TDP-43 transgenic ALS models [38], and chronic gut inflammation has been implicated in AD and FTD pathogenesis [39]. These mechanisms complement the intermediate, context-dependent contributions of GI categories observed in the DSYN priority landscape. Additional DSYN nodes relevant to gastrointestinal dysfunction and gut-microbiome-related pathways are provided in Table A3, illustrating the specific GI and dysbiosis-associated conditions that contribute to the multimorbidity architecture described in this section.

#### 3.7.4. Antecedent Traumatic Brain Injury and Structural Neurological Insult

DSYN nodes related to traumatic brain injury (TBI), skull injury, and post-traumatic syndromes map to Neurological and Sensory (EET) ontology categories and correspond to robust epidemiological findings linking TBI to FTD and AD risk. Traumatic injury can trigger neuroinflammatory cascades, axonal damage, tau and TDP-43 deposition, and chronic neurodegenerative processes [40,41,42]. Although TBI-related DSYN nodes appear across all three diseases, their prominence is strongest for FTD in alignment with its known vulnerability to frontotemporal network degeneration. These associations bolster the interpretation that structural and inflammatory stressors contribute to the multimorbidity architecture, consistent with intermediate DSYN priority patterns for Sensory/EET systems.

#### 3.7.5. Neuropsychiatric Comorbidity and Serotonergic Dysregulation

Several DSYN nodes reflect neuropsychiatric disturbances—including depression, anxiety, agitation, behavioral disinhibition, and mood dysregulation—and map to the Other/Neuro-Psych ontology category. These comorbidities are common in AD and FTD and are increasingly recognized along the ALS–FTD spectrum [6,7,22]. Prior analyses indicated that certain serotonergic pathways may be disproportionately associated with FTD, consistent with clinical evidence showing reduced serotonergic tone in FTD and related behavioral variants. Although node-level granularities are not detailed here, these patterns underscore the prominence of neuromodulatory circuits that co-occur with, and may interact bidirectionally with, systemic multimorbidity processes identified in the DSYN-based analyses.

Taken together, these DSYN themes demonstrate that the 13-category ontology captures not only high-level multimorbidity structure but also specific mechanistic patterns reflected in the literature-derived neuropsychiatric comorbidities. These themes complement the quantitative analyses presented earlier and provide biologically interpretable pathways through which metabolic, inflammatory, neuropsychiatric, cardiovascular, and toxin-related processes may converge or diverge across AD, ALS, and FTD.

### 3.8. PHSU Patterns as a Pharmacological Perspective on Multimorbidity

PHSU analyses provided a pharmacological lens on the DSYN multimorbidity architecture. Many high-importance PHSU nodes shared across diseases or positioned near intersection-like regions corresponded to anti-inflammatory, antioxidant, anxiolytic, neuromodulatory, or cardiometabolic agents. Representative PHSU nodes and their relative proximity patterns in CompositeView are summarized in Table A4, providing node-level support for the pharmacological themes described here. Examples include traditional or natural products such as *Magnolia officinalis* bark extract, *Atropa belladonna* (Belladonna), *Justicia adhatoda* leaf extract, *Kaempferia galanga* root extract, Jobelyn (*Sorghum bicolor*), and Eucalyptus extracts [43,44,45,46,47,48,49]. These substances have reported anti-inflammatory, antioxidant, neuromodulatory, anxiolytic, or membrane-stabilizing properties and therefore naturally map to Metabolic, Immune, and Other/Neuro-Psych axes.

PHSU nodes closer to ALS frequently included cardiotropic or toxin-related compounds, such as specific inotropic agents and toxin-modulating substances [50,51,52]. This pattern echoes the ALS-specific toxin and cardiovascular DSYN signatures. Overall, PHSU findings are concordant with DSYN-based multimorbidity axes and suggest that existing pharmacological strategies already partially target these systems, even if originally developed within disease-specific silos.

## 4. Discussion

In this section, we synthesize the semantic multimorbidity findings across AD, ALS, and FTD, contextualize shared and disease-specific vulnerability patterns, and discuss implications for multimorbidity theory, validation, and future translational investigation.

### 4.1. A Shared Metabolic and Immune Multimorbidity Architecture

This study leverages a large-scale semantic network framework to characterize multimorbidity architecture across Alzheimer’s disease (AD), amyotrophic lateral sclerosis (ALS), and frontotemporal dementia (FTD). While these conditions differ markedly in clinical presentation and neural system involvement, the DSYN and PHSU analyses presented here reveal a deeper systems-level organization that transcends traditional disease boundaries. Rather than existing as isolated disorders, AD, ALS, and FTD appear embedded within overlapping networks of metabolic imbalance and chronic inflammation, accompanied by prominent neurobehavioral processes that co-occur with systemic vulnerability. Together, these patterns suggest a shared substrate capable of shaping disease susceptibility, phenotype expression, and progression across the neurodegenerative spectrum [4,8,9].

Across DSYN hierarchies, intersections, and enrichment analyses, a consistent finding is the emergence of a metabolic–immune axis as the dominant multimorbidity core shared across AD, ALS, and FTD. This axis aligns with the extensive experimental and clinical literature demonstrating that mitochondrial stress, impaired glucose and lipid regulation, systemic inflammation, and microglial priming frequently precede overt neurodegeneration [26,32,39]. Our results provide a computational framework in which these processes are not treated as parallel or independent risk factors; instead, they appear jointly embedded within a shared, literature-defined disease neighborhood that reflects coupled metabolic and immune dysregulation.

### 4.2. Neurobehavioral Processes as Coupled Features of Multimorbidity

Neuropsychiatric and neurobehavioral processes are also prominently represented across disease-specific and intersection contexts, reflecting their close coupling to systemic multimorbidity. However, these processes should be interpreted as co-occurring and interacting features—encompassing both true comorbidities and disease-adjacent manifestations—rather than as independent core drivers of multimorbidity. Their prominence highlights the bidirectional interplay between metabolic, immune, and neurobehavioral systems, consistent with clinical and neurobiological evidence linking mood, cognition, and behavior to inflammatory and metabolic states [6,7,22].

This framing is particularly important in the literature-derived analyses, where disease-defining features and comorbid conditions may coexist within semantic neighborhoods. By explicitly distinguishing coupled neurobehavioral processes from the metabolic–immune core, the present framework avoids over-attributing causal primacy while preserving the biological relevance of neuropsychiatric comorbidity in neurodegenerative disease.

### 4.3. Disease-Specific Vulnerability Profiles and the Bridging Role of FTD

Importantly, the multimorbidity architecture identified here is not uniform across diseases. AD, ALS, and FTD maintain distinct vulnerability profiles that shape how shared systemic stressors are expressed. AD exhibits strong specificity for cardiovascular and vascular–metabolic risk factors, consistent with the established contribution of cerebrovascular integrity to cognitive decline [4,34]. ALS, in contrast, shows heightened association with environmental toxins and neuromuscular pathways, reflecting its susceptibility to exposure-related injury and motor-system stress [12,28].

FTD occupies an intermediate position along this spectrum, with metabolic and neurobehavioral signatures disproportionately represented in multimorbidity intersections. This positioning supports the interpretation of FTD as a connective or “bridge” phenotype linking predominantly cortical neurodegeneration (shared with AD) and motor-system vulnerability (shared with ALS), consistent with molecular and clinical evidence from the ALS–FTD continuum [2,3,23]. This bridging role may help explain why FTD-spectrum processes—such as serotonergic dysregulation, frontal network vulnerability, and behavioral changes—intrude into both ALS and AD clinical spaces.

### 4.4. Intermediate Systems and Multimorbidity Modulation

Beyond the shared metabolic–immune axis, the DSYN priority landscape highlights a set of intermediate systems—including gastrointestinal, endocrine, hematological, hepatic, and sensory pathways—that contribute in context-dependent ways. These systems do not dominate multimorbidity structure independently but may act as modulators or amplifiers through which systemic stressors are integrated. For example, gut dysbiosis may potentiate inflammatory cascades, endocrine disruption may alter metabolic load or stress-axis dynamics, and sensory or environmental interfaces may influence exposure-related vulnerability [10,11]. Together, these findings suggest that a multimorbidity structure reflects layered system interactions rather than additive disease burden alone.

This layered organization suggests that multimorbidity arises from interactions among core systemic processes and secondary organ systems rather than from single dominant pathways. Such interactions may help explain heterogeneity in disease presentation and progression, even among individuals sharing similar molecular pathology.

### 4.5. Preclinical Vulnerability Versus Disease-Associated Comorbidity

An important interpretive consideration is whether the shared metabolic and immune architecture identified here reflects preclinical vulnerability versus disease-associated comorbidity captured in the literature. Because SemNet-derived associations quantify semantic relatedness rather than temporal sequence, the present framework cannot distinguish antecedent risk states from downstream comorbidity or complication. However, DSYN nodes in this analysis encompass risk factors, antecedent conditions, and disease-adjacent phenomena, suggesting that the shared metabolic–immune structure likely reflects a mixture of upstream vulnerability and downstream systemic involvement. Disambiguating these temporal relationships will require integration with longitudinal clinical datasets and in vivo biomarkers.

### 4.6. Implications for Validation and Multimodal Integration

Within this context, the alignment between DSYN and PHSU patterns further supports the relevance of system-level multimorbidity. Shared pharmacological neighborhoods cluster around compounds with anti-inflammatory, antioxidant, neuromodulatory, and cardiometabolic properties—mechanisms that mirror the DSYN-derived architecture [15,53]. While these observations do not imply therapeutic efficacy, they suggest that existing pharmacological strategies already engage portions of shared multimorbidity space.

An important next step is integrating in vivo biomarkers to validate and refine this semantic framework. Quantitative MRI modalities—including diffusion MRI, quantitative susceptibility mapping (QSM), and other advanced imaging metrics—offer a means to test whether individuals with greater metabolic–immune multimorbidity burden exhibit convergent tissue-level phenotypes across AD, ALS, and FTD, and whether FTD occupies an intermediate imaging position consistent with its bridging role. Such multimodal integration could provide a critical bridge between literature-derived multimorbidity architecture and clinical validation [54,55,56].

### 4.7. Limitations

This work has several limitations. DSYN nodes may conflate disease-defining manifestations with comorbid conditions, and HeteSim-based rankings reflect semantic relatedness rather than causal relationships. The ontology, while harmonized and mechanistically motivated, imposes discrete categories on inherently complex biological systems. PHSU analyses are interpretive and depend on the existing pharmacological literature, which may be unevenly distributed across diseases and therapeutic classes.

SemNet 2.0 also carries limitations inherent to literature-based inference. Although the underlying knowledge graph is constructed from more than 35 million PubMed abstracts, the ranking framework is unsupervised and reflects patterns present in the published literature, including biases in research focus, reporting, and terminology. In particular, the substantially larger literature on AD relative to ALS or FTD may influence observed multimorbidity architectures. As such, absence or attenuation of associations should not be interpreted as biological absence. Finally, literature-derived semantic associations do not encode temporality; therefore, preclinical vulnerability versus disease-associated comorbidity cannot be distinguished without longitudinal clinical validation. Notably, prior SemNet 2.0 studies have generated mechanistic and pharmacological hypotheses that were later supported by experimental or clinical evidence [15,17,18,53,57], underscoring the utility of this approach for hypothesis generation while emphasizing the need for independent validation.

## 5. Conclusions

This study presents a systems-level framework for characterizing multimorbidity across Alzheimer’s disease (AD), amyotrophic lateral sclerosis (ALS), and frontotemporal dementia (FTD) using literature-derived semantic networks grounded in DSYN and PHSU representations. Across disease-specific and intersection analyses, we identify a convergent multimorbidity architecture structured around a shared metabolic and immune core, accompanied by intermediate systems that modulate disease expression and distinct disease-specific vulnerabilities.

By situating AD, ALS, and FTD within overlapping networks of systemic dysfunction rather than isolated disease entities, this work provides a unifying perspective on neurodegenerative multimorbidity. The resulting priority landscape offers a principled, hypothesis-generating foundation for multimorbidity-aware investigation, including future biomarker discovery and cross-disease therapeutic exploration, supporting translational efforts grounded in integrated, systems-focused approaches.

## Figures and Tables

**Table 1:** Overall importance rankings for the 13 biological ontology categories. Lower rank indicates higher relative importance. Rankings are shown for each disease. The integrative ranking was computed using a Borda aggregation across AD, ALS, and FTD category ranks (i.e., categories were ordered by the sum of their disease-specific ranks; ties were broken by lower median rank, then lower minimum rank), preserving the ordinal nature of rank data.

Systemic Domain	AD	ALS	FTD	Integrative (Borda)
Autoimmune, Immunological, Infectious Disorders	2	1	2	1
Metabolic Dysfunction and Mitochondrial Impairment	1	2	3	2
Cardiovascular Pathology or Dysfunction	3	9	4	3
Gastrointestinal or Gut–Brain Axis Disorders	4	8	5	4
Endocrine and Hormonal Imbalances	5	6	6	5
Hematological Dysfunction	6	7	7	6
Other/Neuropsychiatric Disorders	7	13	1	7
Kidney Dysfunction	8	12	9	11
Liver Dysfunction	9	4	10	8
Dermatological	10	11	12	13
Eyes, Ears, Throat (EET)	11	3	13	9
Environmental Toxins	12	5	11	10
Orthopedic and Muscle	13	10	8	12

## Data Availability

The SemNet 2.0 and CompositeView software is open-source and available at https://github.com/pathology-dynamics, accessed on 12 February 2026.

## Appendix A

### Appendix A.1

**Table A1:** Representative diabetes-related DSYN source nodes shared across Alzheimer’s disease (AD), amyotrophic lateral sclerosis (ALS), and frontotemporal dementia (FTD). HeteSim scores for each disease are shown for selected nodes spanning type 1 diabetes, type 2 diabetes (including early- and late-onset forms), maturity-onset diabetes of the young (MODY), childhood diabetes, and other metabolic diabetes subtypes.

Source Node Name	AD	ALS	FTD
Type 1 diabetes mellitus with nephropathy	0.756	0.828	0.997
Type 2 diabetes mellitus in remission	0.630	0.662	0.630
Type 3 diabetes	0.690	0.618	0.943
MODY type 8 with exocrine dysfunction	0.989	0.995	0.992
MODY type 10	0.979	0.988	0.977
Maturity-onset diabetes mellitus in youth	0.577	0.741	0.878
Early-onset type 2 diabetes	0.313	0.220	0.249
Childhood diabetes mellitus	0.997	0.964	0.886
Familial diabetes insipidus	0.862	0.957	0.942
Pancreatogenous diabetes	0.328	0.693	0.917
Mitochondrial diabetes	0.376	0.769	0.840

**Table A2:** Cardiovascular-related DSYN source nodes shared across Alzheimer’s disease (AD), amyotrophic lateral sclerosis (ALS), and frontotemporal dementia (FTD). Nodes correspond to hypertension, hypercholesterolemia, and obesity subclasses, and representative HeteSim scores for each disease are shown.

Source Node Name	AD	ALS	FTD
Sporadic primary pulmonary hypertension	0.892	0.946	0.963
Familial primary pulmonary hypertension	0.951	0.935	0.892
Idiopathic pulmonary hypertension	0.545	0.678	0.780
Pulmonary arterial hypertension	0.790	0.687	0.708
Venous hypertension	0.629	0.406	0.671
Secondary hypertension	0.585	0.544	0.635
Neurogenic hypertension	0.732	0.591	0.485
Hypercholesterolemia, autosomal recessive	0.953	0.982	0.976
Hypercholesterolemia	0.859	0.636	0.554
Primary hypercholesterolemia	0.702	0.667	0.414
Secondary hypercholesterolemia	0.919	0.831	0.505
Homozygous familial hypercholesterolemia	0.424	0.844	0.876
Familial hypercholesterolemia (heterozygous)	0.878	0.713	0.407
Polygenic hypercholesterolemia	0.665	0.481	0.813
Obesity	0.861	0.525	0.364
Lifelong obesity	0.861	0.674	0.407
Adult-onset obesity	0.658	0.653	0.736
Moderate obesity	0.600	0.566	0.744

**Table A3:** Representative gastrointestinal-related DSYN source nodes shared across Alzheimer’s disease (AD), amyotrophic lateral sclerosis (ALS), and frontotemporal dementia (FTD). These nodes reflect conditions associated with gut microbiome dysbiosis, gastrointestinal inflammation, or microbial imbalance. HeteSim scores for each disease are provided.

Source Node Name	AD	ALS	FTD
Neonatal gastrointestinal disorder	0.999	0.983	0.980
Upper gastrointestinal disorders	0.948	0.823	0.854
Gastrointestinal infection	0.790	0.780	0.833
Gastrointestinal candidiasis	0.803	0.925	0.798
Gastrointestinal anthrax	0.821	0.934	0.795
Gastrointestinal inflammation	0.814	0.696	0.733
Functional gastrointestinal disorders	0.824	0.645	0.666
Gastrointestinal diseases	0.822	0.596	0.483

**Table A4:** Representative PHSU (pharmacological substance) nodes shared across Alzheimer’s disease (AD), amyotrophic lateral sclerosis (ALS), and frontotemporal dementia (FTD). Shown are composite HeteSim scores (arithmetic mean of AD, ALS, and FTD scores) and qualitative proximity interpretations based on CompositeView positioning.

Source Node Name	Composite Score	CompositeView Proximity
Gallein	0.442	Closer to FTD
Non-nucleoside reverse transcriptase inhibitors	0.561	Closer to FTD
Dihydrotestosterone 17-bromoacetate	0.432	Closer to FTD
11-ketoprogesterone	0.480	Closer to FTD
Senicapoc	0.754	Closer to FTD
Streptococcus vaccine	0.393	Closer to FTD
Andexanet alfa	0.542	Closer to FTD
Z-VAD-DCB	0.467	Closer to FTD
Ultragel AcA-34	0.348	Closer to FTD
Rp-CPT-cAMPS	0.218	Closer to FTD
8-pCPT-cGMP	0.350	Closer to FTD
AZD7442	0.305	Closer to FTD
Yeast concentrate	0.543	Closer to FTD
Addictive analgesics	0.358	Closer to FTD
Zolimid	0.997	Center
Stealth liposomal cisplatin	0.992	Center
Gnetin C	0.959	Center
2-formylpyridine thiosemicarbazone	0.960	Center
Justicia adhatoda leaf extract	0.965	Center
Antibacterial, topical	0.965	Center
Dihydrohomofolic acid	0.965	Center
Rubiscolin 5	0.965	Center
Prunus avium extract	0.965	Center
Eucalyptus gum extract	0.961	Center
Jobelyn (*Sorghum bicolor* extract)	0.965	Center
Kaempferia galanga root extract	0.961	Center
Magnolia officinalis bark extract	0.990	Center
Belladonna (*Atropa belladonna*) extract	0.878	Center
Antihuman endoglin immunotoxin	0.965	Center
